# A generalized pustular psoriasis flare after CoronaVac COVID-19 vaccination: Case report

**DOI:** 10.34172/hpp.2021.32

**Published:** 2021-05-19

**Authors:** Nahide Onsun, Gökhan Kaya, Bengisu Güçkan Işık, Begüm Güneş

**Affiliations:** Department of Dermatology and Venereology, Bezmialem Vakif University, Istanbul, Turkey

**Keywords:** CoronoVac, COVID, Psoriasis

## Abstract

CoronoVac is a non-viable vaccine for severe acute respiratory syndrome coronavirus 2 (SARSCoV-2). Nowadays, there has been vaccination program for at-risk groups and older adults in Turkey. We here present 72-year-old male psoriasis patient who developed generalized pustular psoriasis flare after administration of CoronoVac. The COVID-PCR test was negative and investigations for flare etiology were all normal. He was first (to the best of our knowledge) psoriasis patient who developed an erythrodermic flare after the first dose of CoronaVac vaccine.

## Introduction

CoronaVac (Sinovac Biotech) is a non-viable vaccine for SARS-CoV-2. Phase 3 trials have been conducted in Turkey. A nationwide vaccination program for at-risk groups and older adults is ongoing. As the number of vaccinated people increases, we will learn more about adverse reactions. We here present the first (to the best of our knowledge) psoriasis patient who developed an erythrodermic flare after the first dose of CoronaVac vaccine.

## Case Report

**Figure 1:**
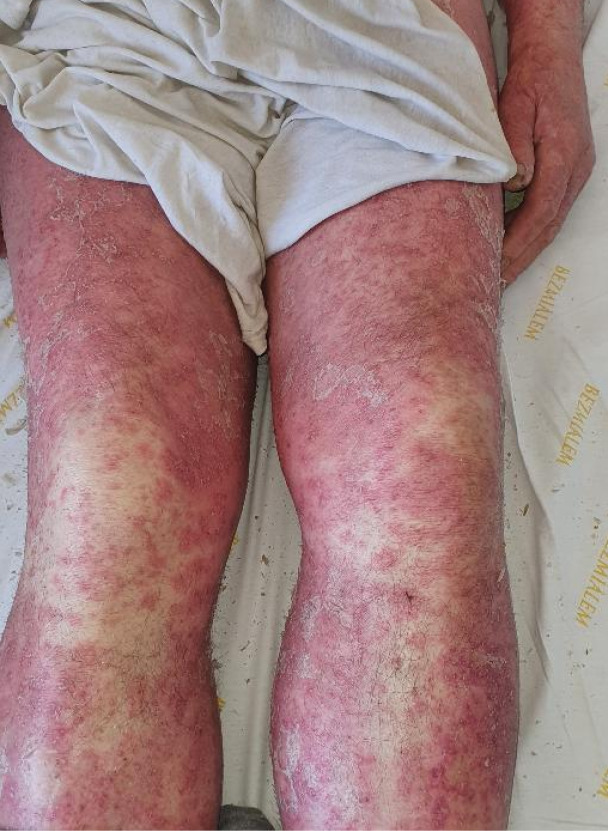
Diffuse erythematous scaly plaques with coalescing pustules and intact skin areas on the lower limbs.

**Figure 2:**
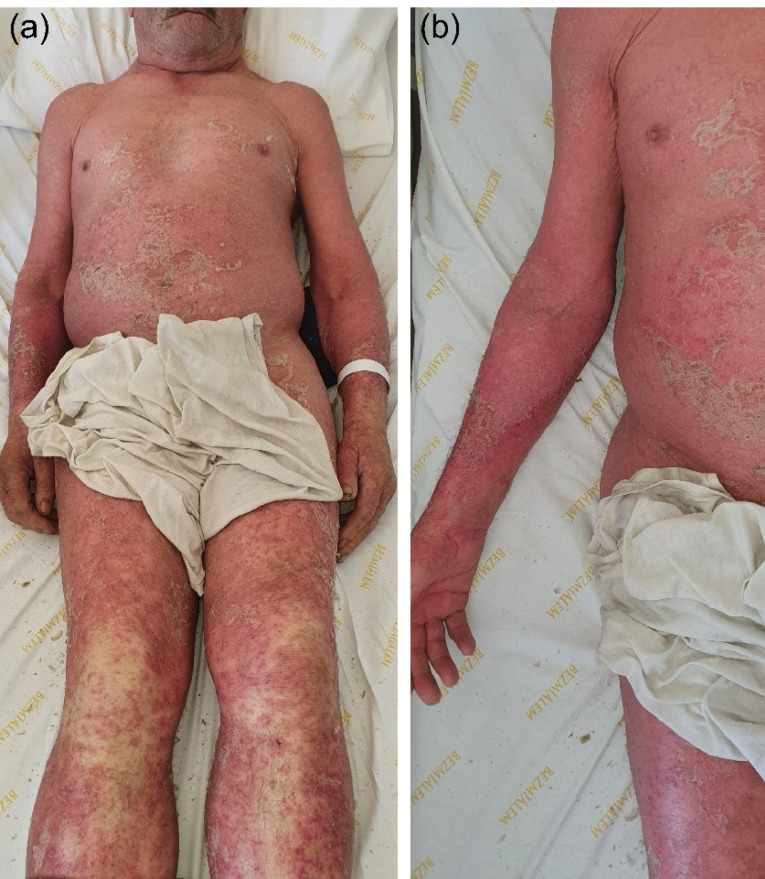
(a, b) Diffuse erythematous scaly plaques with coalescing pustules over the entire body.

A 72-year-old male was referred by our emergency department with diffuse erythema, desquamation, fever, and a poor general condition. He had a history of plaque psoriasis, but used only topical treatments. He had a recent history of prerenal acute injury caused by dehydration and had been taking indapamide for many years to treat hypertension. He had received the CoronaVac vaccine 4 days before (February 15, 2021; 3:00 pm) the rash appeared. Physical examination revealed diffuse erythema, desquamation, and coalescing pustules over the entire body (Figures 1 and 2). Initial laboratory investigation revealed elevated levels of acute-phase reactants, confirming disease activation. The COVID-PCR test was negative and the peripheral blood smear was normal. He had no history of a malignancy; the tumor marker tests were negative. Histopathology was also compatible with generalized pustular psoriasis. He was thus diagnosed with a generalized pustular psoriasis exacerbation associated with CoronaVac administration.

We commended acitretin at 25 mg/d but this had no effect. Intravenous infliximab infusion at 5 mg/kg afforded a complete response.

He voluntarily confirmed his willingness to be published with his clinical records.

## Discussion

Immune system-mediated diseases including psoriasis increase the susceptibility to infections partly because most treatments are immunosuppressive and partly because of the nature of the diseases per se.^1^ This compromises patient prognosis and survival; specific vaccination protocols should be considered.^2^ Genetic factors may trigger flaring of pre-existing psoriasis or induce de novo disease. Recent studies have revealed some causal links between certain vaccinations and psoriasis. Hung et al^3^ reported a case of new-onset guttate psoriasis after intravesical BCG administration to treat bladder cancer; several cases of psoriasis flares after H1N1 influenza vaccination were described bySbidian et al.^4^ Yoneyama et al. presented a case exhibiting psoriasis exacerbation after pneumococcal polysaccharide vaccination.^5^

Farkas et al^6^ found that vaccines may activate the plasmacytoid and dermal myeloid dendritic cells that play roles in the inflammatory psoriasis cascade. These dendritic cells connect environmental factors to T lymphocytes. The cells express Toll-like receptors of subtypes 7, 8, and 9; after binding of antimicrobial peptide LL37, the cells release the inflammatory mediators IL-6, IL-12, TNF-α, and TGF-β. These mediators in turn induce T cells to differentiate into T_h1_ and T_h17_ cells, which then release the cytokines TNF-α, IFN- γ, IL-12, IL-22, and IL-23 that cause psoriatic skin changes.^4,6-8^

COVID-19 vaccines are being rapidly delivered worldwide. The numbers and types of adverse effects will increase, improving our knowledge. We present the first case of CoronaVac-associated generalized pustular psoriasis. Although patients with chronic autoimmune conditions such as psoriasis must of course be vaccinated, they should be carefully monitored.

## Competing interests

None.

## Ethical approval

Informed consent was obtained from the patient for publication of this report.

## Authors’ contributions

NO was involved in the evaluation of patient, design of study, writing original draft, and supervision. GK, BGI and BG were involved in writing and editing of draft. All authors provided feedback and helped shape the manuscript.
